# Supplementary material for: Comprehensive High-Depth Proteomic Analysis of Plasma Extracellular Vesicles Containing Preparations in Rett Syndrome
Source: Biomedicines. 2024 Sep 24;12(10):2172. doi: 10.3390/biomedicines12102172 (PMC11504846; doi:10.3390/biomedicines12102172)
Supplement: Supplementary file 1 [file biomedicines-12-02172-s001.zip › Figure S1_Number of protein detections per mass extracted.pdf]

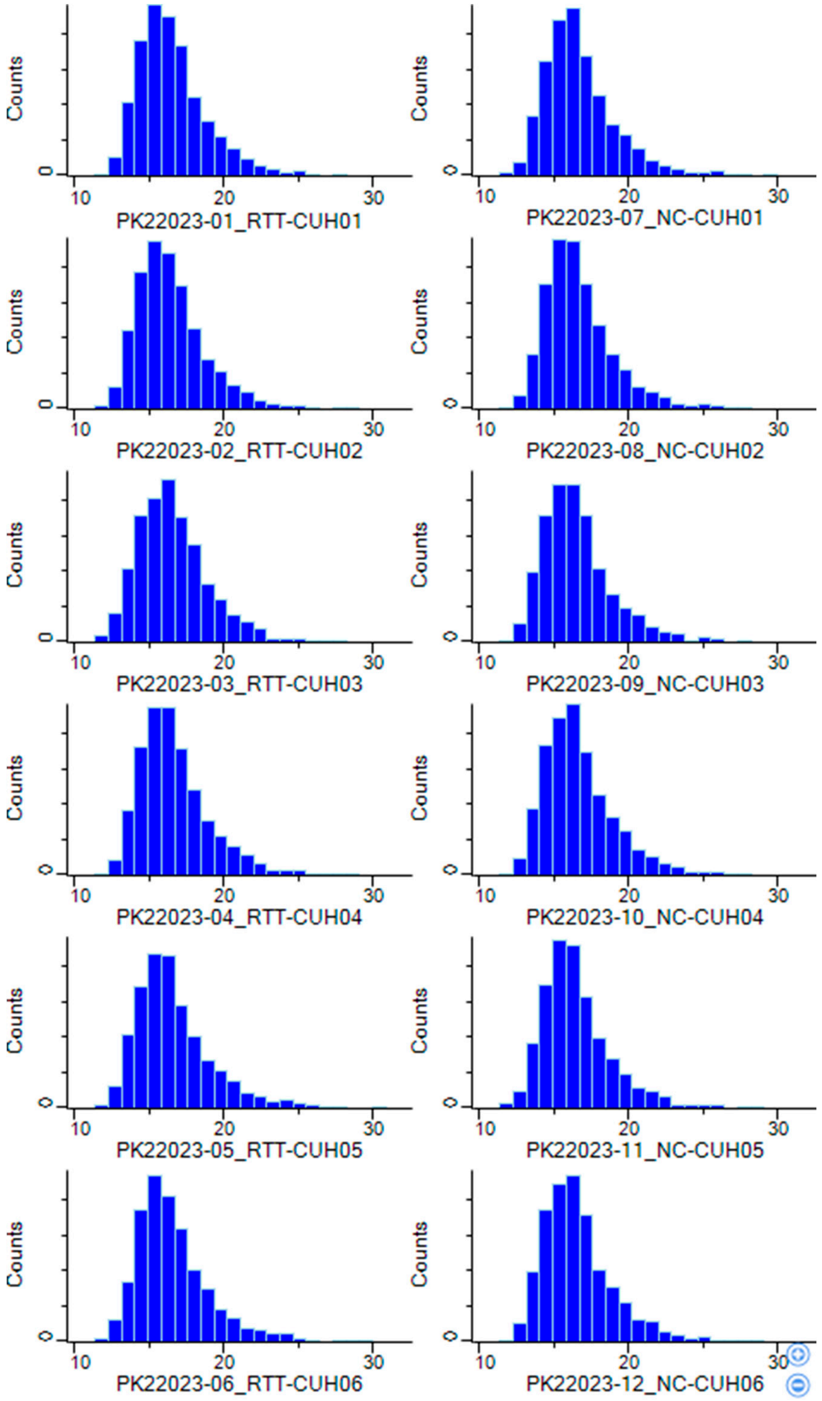

Figure S1. **Number of protein detections per mass extracted**

Histograms for the number of protein detections per mass extracted in the Rett syndrome (RTT) group and the neurotypical developmental control (NC) group. X axis and Y axis indicate log2 value of each protein and the numbers of detected proteins, respectively.
