# Supplementary material for: Comprehensive High-Depth Proteomic Analysis of Plasma Extracellular Vesicles Containing Preparations in Rett Syndrome
Source: Biomedicines. 2024 Sep 24;12(10):2172. doi: 10.3390/biomedicines12102172 (PMC11504846; doi:10.3390/biomedicines12102172)
Supplement: Supplementary file 1 [file biomedicines-12-02172-s001.zip › Figure S2_Correlation of up- and downregulated proteins and CSS.pdf]

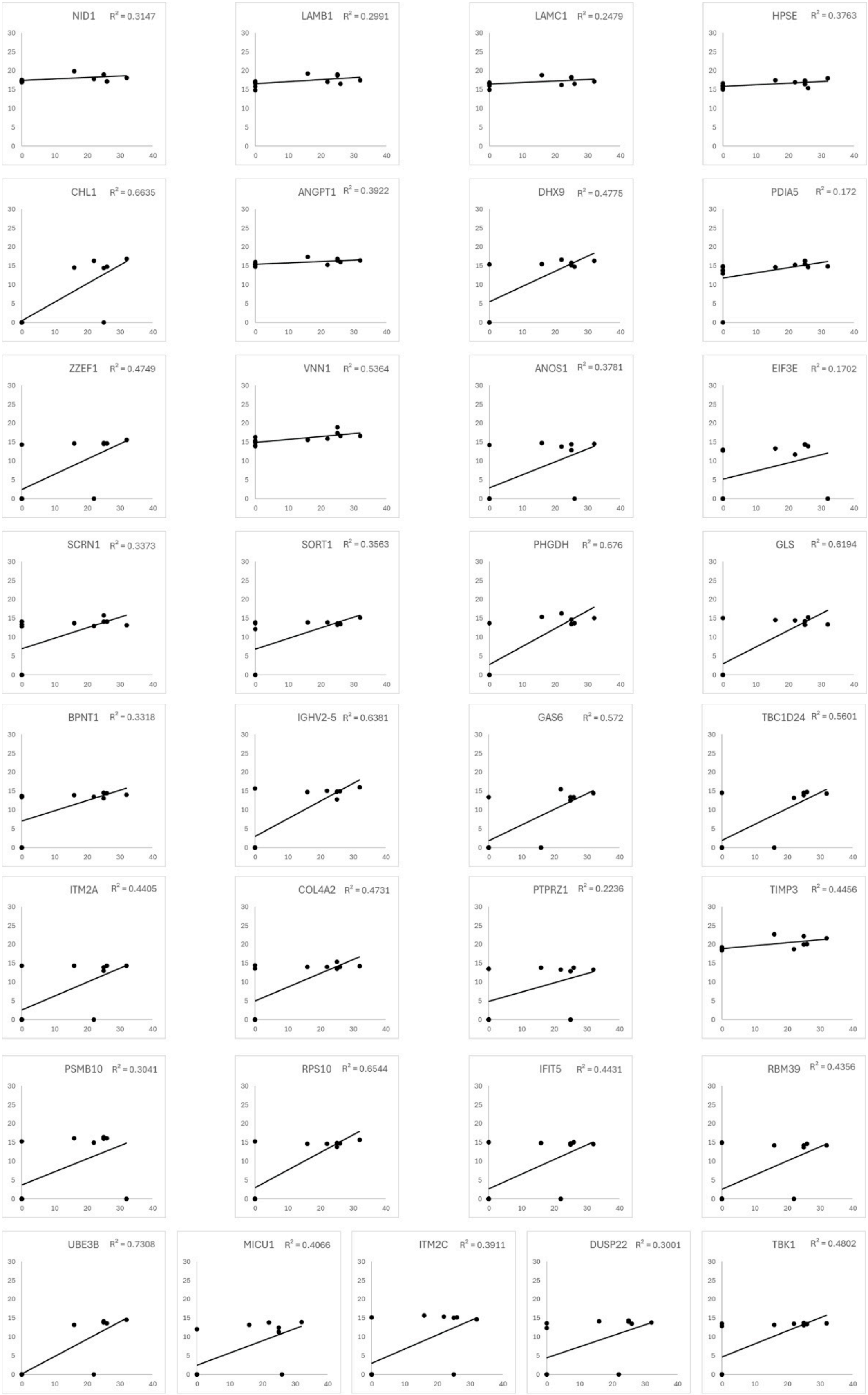
NID1 $R^2 = 0.3147$
LAMB1 $R^2 = 0.2991$
LAMC1 $R^2 = 0.2479$
HPSE $R^2 = 0.3763$
CHL1 $R^2 = 0.6635$
ANGPT1 $R^2 = 0.3922$
DHX9 $R^2 = 0.4775$
PDIA5 $R^2 = 0.172$
ZZEF1 $R^2 = 0.4749$
VNN1 $R^2 = 0.5364$
ANOS1 $R^2 = 0.3781$
EIF3E $R^2 = 0.1702$
SCRN1 $R^2 = 0.3373$
SORT1 $R^2 = 0.3563$
PHGDH $R^2 = 0.676$
GLS $R^2 = 0.6194$
BPNT1 $R^2 = 0.3318$
IGHV2-5 $R^2 = 0.6381$
GAS6 $R^2 = 0.572$
TBC1D24 $R^2 = 0.5601$
ITM2A $R^2 = 0.4405$
COL4A2 $R^2 = 0.4731$
PTPRZ1 $R^2 = 0.2236$
TIMP3 $R^2 = 0.4456$
PSMB10 $R^2 = 0.3041$
RPS10 $R^2 = 0.6544$
IFIT5 $R^2 = 0.4431$
RBM39 $R^2 = 0.4356$
UBE3B $R^2 = 0.7308$
MICU1 $R^2 = 0.4066$
ITM2C $R^2 = 0.3911$
DUSP22 $R^2 = 0.3001$
TBK1 $R^2 = 0.4802$

(a)

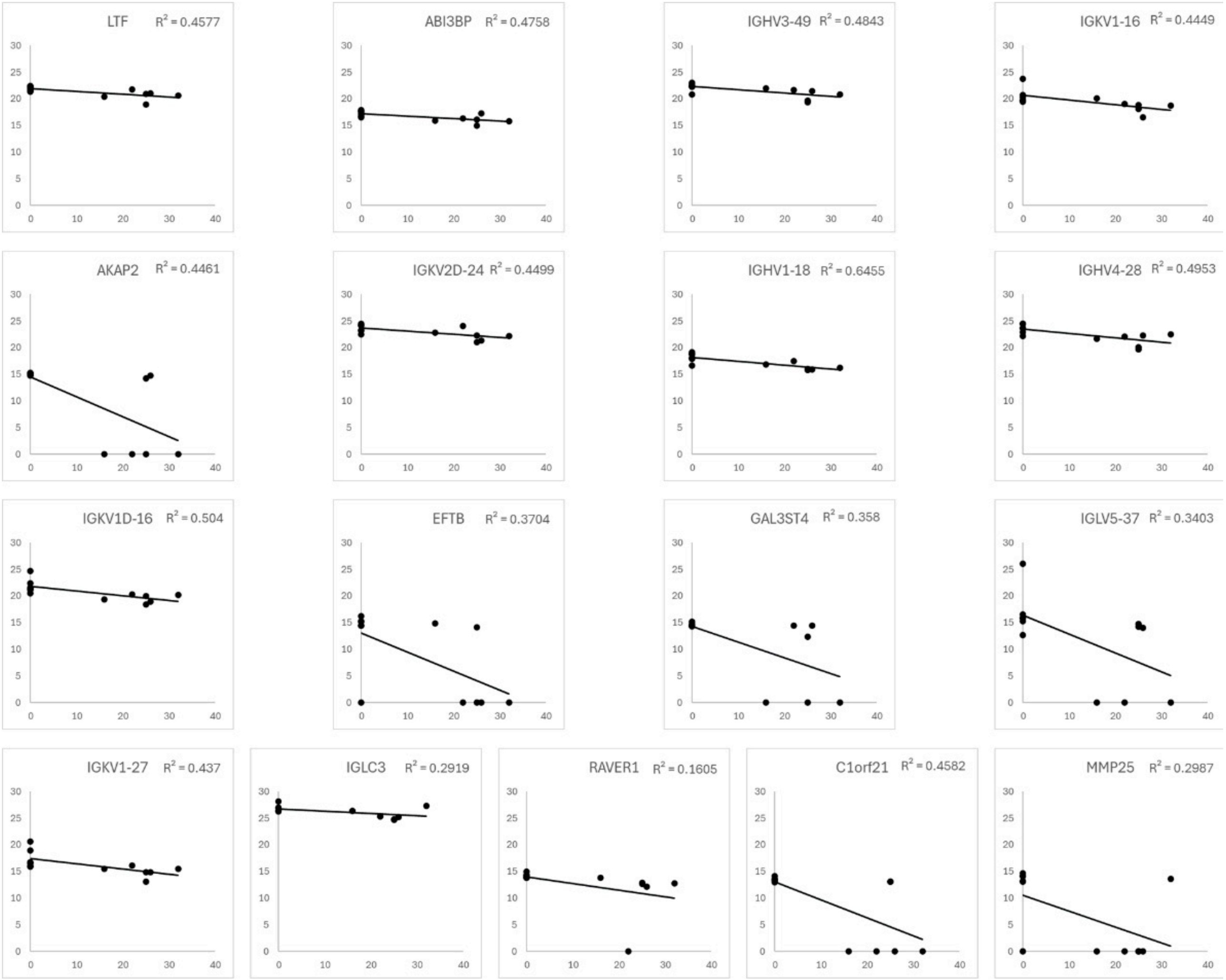

(b)

Figure S2. Correlation of up- and down-regulated proteins and CSS score

On the 33 up- (a) and 17 down-regulated (b) proteins, scatter plots of the log2-transformed protein masses versus the CSS scores in RTT group, with the regression line indicated. The vertical axis represents protein mass, and the horizontal axis represents the CSS score.
